# Behavioral and electroantennogram responses of plum curculio, Conotrachelus nenuphar, to selected noxious plant extracts and insecticides

**DOI:** 10.1093/jis/14.1.90

**Published:** 2014-07-08

**Authors:** A. Gӧkçe, L. L. Stelinski, D. R. Nortman, W. W. Bryan, M. E. Whalon

**Affiliations:** 1 Nigde University, Faculty of Agricultural Sciences and Technologies, Department of Plant Production and Technologies, Nigde, Turkey; 2 University of Florida, Entomology and Nematology Department, Citrus Research and Education Center, 700 Experiment Station Road, Lake Alfred, FL; 3 Michigan State University, Department of Entomology, Pesticide Alternative Laboratory Center for Integrative Plant Systems, East Lansing, MI

**Keywords:** Arctium, Bifora, Humulus, Verbascum, Xanthium, botanical, antifeedant, oviposition deterrent

## Abstract

Behavioral and electroantennogram responses of plum curculio, *Conotrachelus nenuphar* (Herbst) (Coleoptera: Curculionidae), adults were tested for several methanolic plant extracts and organically approved insecticides. Plant extracts were evaluated for their potential as antifeedants or oviposition deterrents. These extract responses were also compared to those elicited by the non-neurotoxic, organic irritant-insecticide kaolin clay. Both sexes of plum curculio exhibited antennal response as measured by electroantennogram, which ranged from 0.2 to 1.1 mV, to plant extracts and the organic irritant/insecticide, with the greatest response to the extract of rough cocklebur, *Xanthium strumarium* L. (1.1 mV). No choice tests were conducted to compare feeding and oviposition by plum curculio on untreated apples or on apples treated with one of the extracts or the insecticide. The insecticide pyrethrum and extracts of *X. strumarium* and greater burdock, *Arctium lappa* L., significantly reduced feeding. Also, pyrethrum *, A. lappa, Humulus lupulus* L. (common hop), *X. strumarium,* and *Verbascum songaricum* Schrenk extracts completely inhibited egg deposition. In no-choice assays, the effects of kaolin clay with incorporated plant extracts on plum curculio feeding and oviposition were monitored as complementary tests. *A. lappa-kaolin, H. lupulus* –kaolin, and *X. strumarium-kaolin* mixtures significantly reduced the feeding of plum curculio compared to the control or kaolin clay alone. Each of the plant extract-kaolin mixtures evaluated, with the exception of *Bifora radians* Bieberstein (wild bishop), completely inhibited plum curculio oviposition as compared to controls.

## Introduction

Plum curculio, *Conotrachelus nenuphar* (Herbst) (Curculionidae: Coleoptera), is a tree fruit pest native to North America east of the Rocky Mountains. It causes economically significant damage in rosaceous tree fruit in the eastern United States, particularly in tart and sweet cherry, pome fruit, and peach ( Chapman 1938 ). It is also known to cause damage to highbush blueberry (Polaravapu et al. 2004). Adults of both sexes feed on fruit by making a small puncture in the skin and eating the flesh underneath. Female plum curculio also make crescent-shaped oviposition scars in the developing fruit, where they lay their eggs. Both of these behaviors reduce the marketability of fruit. As a result, scar tissue and/or developing larvae feeding can cause significant yield loss in commercial orchards, and the legal prohibition or “no worm” policy in Michigan cherries (Hall 1977; USDA-AMS) effectively creates zero tolerance for plum curculio larvae in processed cherries. Today, this 1940’s law has been substantially strengthened by emerging “good agricultural practices” or good agricultural practice marketing standards, along with international infestation export barriers for Upper Midwest tree fruit.

The passage of the Food Quality Protection Act ( FQPA 1996 ) essentially has cancelled the use of key organophosphate insecticides such as azinphos-methyl (AZM) ( US EPA 2008 ), an insecticide that sufficiently penetrates rapidly growing fruit to eliminate larvae while effectively controlling adults and leaving no or very little residue at harvest. In both international and national markets, maximum residue limit (MRL) standards with AZM use were almost never violated, while numerous “reduced-risk” ( US EPA 2012 ) insecticides have triggered rejections in domestic and foreign markets ( Whalon et al. 2011 ). Therefore, today’s Upper Midwest growers, representing 85% of the USA’s tart cherry production (NASS 2012), are seeking effective alternative means to replace AZM while achieving excellent control of internally tunneling larvae.

As AZM is being phased out ( US EPA 2008 ), phosmet (Imidan™) as well as synthetic pyrethroids have been suggested as possible replacements in IPM programs, and its use by many growers in the Upper Midwest is increasing rapidly ( Whalon et al. 2011 ). So far, these and other “replacement” pesticides are typically less effective for plum curculio control and require more sprays or more complicated pesticide use patterns to achieve economic control. More frequent applications result in a higher risk of resistance while negatively impacting the contribution of beneficial organisms to ecological services ( Whalon et al. 2011 ). To date, AZM’s replacement selection has not been a straightforward matter in Upper Midwest stone fruit orchards, and there is a need for additional strategies and tactics to mitigate risks of pest-inflicted damage, breach of good agricultural practice standards, and violation of maximum residue limits in foreign markets.

Conventional stone fruit producers in the Upper Midwest are not alone in the struggle to control plum curculio. Organic producers throughout eastern North America also struggle to control this key pest ( Grieshop et al. 2010 ). The tools available generally are prohibitively expensive, very labor intensive, or impart unacceptable maximum residue limit risks in export markets. Previous work with plum curculio in Upper Midwest organic stone fruit production has introduced “attract and kill” or “push-pull” methods, which rely on marginally effective plant-based kairomones. The “push” in this strategy has primarily been the use of repeated applications of kaolin clay/diatomaceous earth and pyrethrum ( Whalon 2008 ; Grieshop et al. 2010 ). The excessive number of applications of kaolin clay required for effective control leads to increased costs for growers; therefore, the development and adoption of a more effective “push” than kaolin clay could decrease the cost and increase the effectiveness of pyrethrum as the killing agent for organic growers. Aggregating plum curculio with repellents by modifying their behavior may increase their vulnerability to spot treatments of insecticides or even deployment of killing stations composed of baited pyramid traps ( Coombs 2001 ). The availability of a more effective and inexpensive aggregate and kill method would therefore be a useful new tool for Upper Midwest growers, particularly organic producers.

Natural products have been a foundation for plum curculio monitoring ( Garman and Zappe 1929 ; Coombs 2001 ; Leskey et al. 2008 ). Plum essence, an extract of ripe plum fruits, is an attractive kairomone that tree fruit scouts have used to trap and monitor plum curculio ( Whalon 2008 ). Also, organic growers have very few tools other than pyrethrum ( Grieshop et al. 2010 ) for plum curculio and other orchard pests. Therefore, this research explored a range of other plant extracts ( Table 1 ) from plant species known to cause behavioral responses in a variety of insects, including Colorado potato beetle ( *Leptinotarsa decemlineata* (Say.) [Coleoptera: Chrysomelidae]), redbanded leafroller *(Argyrotaenia velutinana* (Walker) [Lepidoptera: Tortricidae]), and obliquebanded leafroller *(Choristoneura rosaceana* (Harris) [Lepidoptera: Tortricidae]) ( Gökçe et al. 2005 , 2006a, 2006b, 2010). All of these plants occur naturally in the USA, so demonstration of their utility to growers could lead to implementation of various organic plant mixtures as a relatively inexpensive addition to plum curculio management. Therefore, the behavioral effects of these compounds were investigated in laboratory settings in comparison with other known (currently used or previously studied) repellents and insecticides discussed above.

**Table 1.**
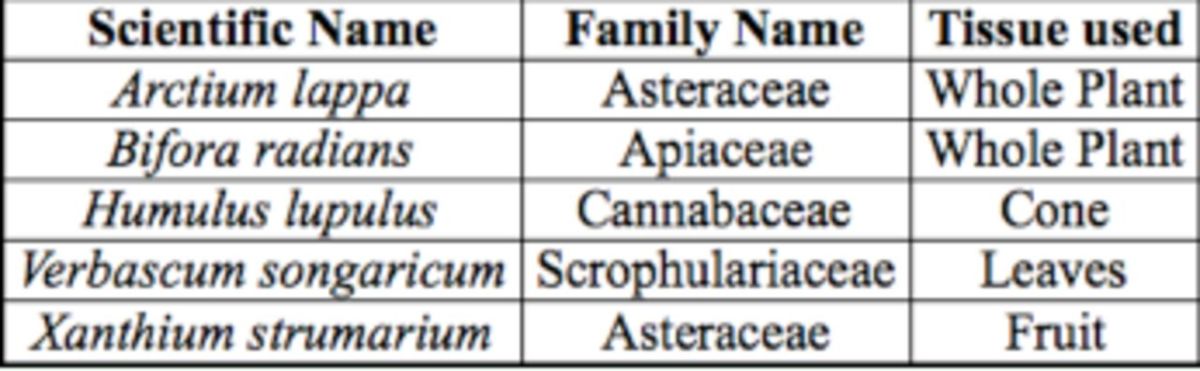
Plants used in behavioral and electroantennogram experiments

The objectives of this study were to determine the effects of selected plant extracts and plum curculio insecticides on (1) plum curculio antennal responses as measured by electroantennogram, (2) feeding and female oviposition behavior, and (3) the combined impact of these plant extracts with kaolin clay on plum curculio female feeding and oviposition behavior.

## Materials and Methods

### Insects

Field-collected, second-generation, Northern strain plum curculio were used in all experiments. Adults were reared at 23 ± 1ºC with a 16:8 L:D photoperiod on thinning apples until used in experiments. Plum curculio adults from this colony were sexed as described by Thomson (1932) and held separately in plastic cages (100 mm × 100 mm × 80 mm). The Northern strain of plum curculio requires adult winter diapause before reproductive maturation occurs, with oviposition commencing the following spring. Thus, they were treated with pyriproxifen (Esteem™), 0.26 gr a.i./L, prior to the experiment in order to break adult winter diapause and promote egg production in the females (Hoffman et al. 2007). Apples were dipped into the pyriproxifen solution for 10 sec and then transferred into a fume hood. Apples were then transferred into plastic cages, after which five male and the same number of female plum curculio adults were introduced. They were incubated at the above conditions for 48 hr and allowed to mate.

### Experimental materials

The sources of plant extracts used for electroantennogram recordings and behavioral assays are described in Table 1 . Plant extracts were collected in Tokat, Turkey, and prepared as described in Gökçe et al. (2005) . All source plant material was dried at room temperature for seven days and subsequently macerated with a grinder (M 20 IKA Universal Mill, IKA Group, www.ika.com ), transferred into sealed glass jars, and stored in the dark at 15 ± 2°C until use. Samples of 50 g were placed into 1000 mL Erlenmeyer flasks with 500 mL of methanol (Sigma-Aldrich, www.sigmaaldrich.com ). These were then placed on a horizontal shaker (HS 260 Basic, IKA Group ) (120 oscillations/min) and shaken for 24 hr. The suspension was filtered through two layers of cheesecloth, and excess methanol was evaporated in a rotary evaporator (RV 05 Basic 1B, IKA Group) at 32 ± 2ºC. The resulting residue was weighed and eluted with acetone to yield 1% (w/v) plant suspensions.

The commercial formulations of pyrethrum (Pyganic™), azadirachtin (Neemix™), and kaolin clay (Surround™) were used as chemical and non-chemical insecticide standards in both electroantennogram and behavioral tests. Pyrethrum, azadirachtin, and kaolin clay were diluted with water to yield 0.25, 0.16, and 56.9 gr a.i./L rates.

### Electroantennogram recordings

The electroantennogram system and test protocols have been described previously ( Stelinski et al. 2003 ). In brief, the electroantennogram system used was a data acquisition interface board (Type IDAC-02) and a universal single-ended probe (Type PRS-1) from Syntech ( www.syntech.nl ). The recording and indifferent electrodes consisted of silver wire in 10 μL glass micropipettes filled with 0.5 M KCl. Data were recorded onto a Gateway 2000 (P-75) computer ( www.gateway.com ) equipped with an interface card and software (PC-EAG version 2.4) from Syntech. Electroantennogram cartridges were made by pipetting 0.25 mg of each plant extract in 25 μL of acetone or 0.05 mg pyrethrum, 0.04 mg azadirachtin, or 0.16 mg kaolin clay in 25 μL of water onto 1.4 x 0.5 cm strips of Whatman No. 1 filter paper. Filter papers were held for 15 min in a fume hood prior to testing to allow for solvent evaporation. Subsequently, treated strips were inserted into disposable glass Pasteur pipettes. Electroantennogram responses were measured as the maximum amplitude of depolarization elicited by 1-mL puffs of air through electroantennogram cartridges using live-insect preparations.

Insects were mounted on a wax-filled, 3.5-cm diameter Petri dish with a clay strip (10 x 3 mm) placed over their thorax and abdomen. The recording electrode was positioned over the tip of the antennal club while the reference electrode was inserted into the head near the base of the antenna. For each chemical assayed, electroantennograms were recorded from 10 insects of each sex. Control stimulations (using filter paper impregnated with 20 **^** L of acetone solvent or water) were delivered before and after each stimulus presentation. Two puffs of each volatile treatment and control spaced 12 sec apart were applied to the antenna to yield duplicate depolarization amplitudes for each replicate beetle. The experiment was conducted in a randomized complete block design with chemical odor and plum curculio sex as factors.

### Behavioral tests

Assays were designed to test the effect of chemicals on feeding and oviposition behavior. Thinning apples that were similar in size (Empire variety, 35 ± 5 mm in diameter), shape, and color were used in all bioassays. Apples were prepared by rinsing with a 0.1% bleach solution in deionized water to remove potential insecticide and/or dust residue and then left to dry in a fume hood for 60 min. Suspensions of 1% (w/v) concentrations of each plant extract were prepared, along with separate solutions of field-recommended rates for insecticides (0.25, 0.16, and 56.9 gr a.i./L for pyrethrum, azadirachtin, and kaolin clay, respectively). Apples were subsequently treated by dipping them individually in 50 mL of plant-extract suspensions or 50 mL of insecticide solutions for 10 sec before drying in a fume hood for 20 min. In the negative control, apples were dipped in acetone for 10 sec and otherwise handled identically to the other treatments.

Beetle response was evaluated in no-choice assays using 100 mm × 100 mm × 80 mm plastic cages. Two apples were attached to the cage bottom using a small drop of hot glue before 10 plum curculio adults (1:1 sex ratio) were transferred into each cage. The cages were incubated at 28 ± 2ºC with a 16:8 L:D photoperiod for 72 hr. After the incubation period, apples were removed and assessed for oviposition and feeding scars. The experiment was arranged as a randomized block design with three replicates. Each block consisted of eight treatments with an associated control.

A complementary experiment was conducted to examine the effects of each plant extract when combined with kaolin clay on plum cur-culio behavior. Suspensions of plant extracts were admixed with kaolin clay by combining 1% (w/v) concentration solutions of extracts with the kaolin clay at its recommended field rate of 56.9 gr a.i./L. Apples were dipped into the plant extract-kaolin clay mixture for 10 sec and left to dry in a fume hood for 20 min. Two apples were again glued to a cage, and 10 plum curculio adults were introduced into each cage, as described above. Oviposition marks and feeding scars were assessed after 72 hr of incubation, as described previously. The experiment was arranged as a randomized block design with three replicates.

### Statistical analyses

Antennal responses of plum curculio were subjected to analysis of variance (ANOVA). Following verification of significant ANOVA, Tukey’s multiple comparison tests were conducted to determine differences between treatment means (Minitab16 statistical software 2010, www.minitab.com ). Two-sample *t* -tests (Minitab) were performed to determine whether beetle response to plant extracts varied significantly between the sexes. The data gathered from both feeding and oviposition tests were subjected to ANOVA followed by the Tukey’s multiple comparison tests to examine differences between treatments (Minitab).

## Results

Electroantennogram depolarizations recorded from male plum curculio were greater than those recorded from females in response to odors from *X. strumarium* and *H. lupulus* (F = 11.81; df = 1, 8; *P <* 0.01) ( Table 2 ). Odors from azadirachtin and kaolin clay produced the lowest responses ( Table 2 ). There was no difference in responses elicited by odors from *V. songaricum* , *B. radians* , *A. lappa* extracts, and pyrethrum ( Table 2 ). Electroantennogram responses of females to the treatments were not as distinct as those seen in males. *X. strumarium* and *H. lupulus* extracts caused the highest depolarizations, which were significantly different from the control ( *P* < 0.05) ( Table 2 ). Odors from all of the other treatments tested were not significantly different from the control ( *P* > 0.05) ( Table 2 ).

**Table 2.**
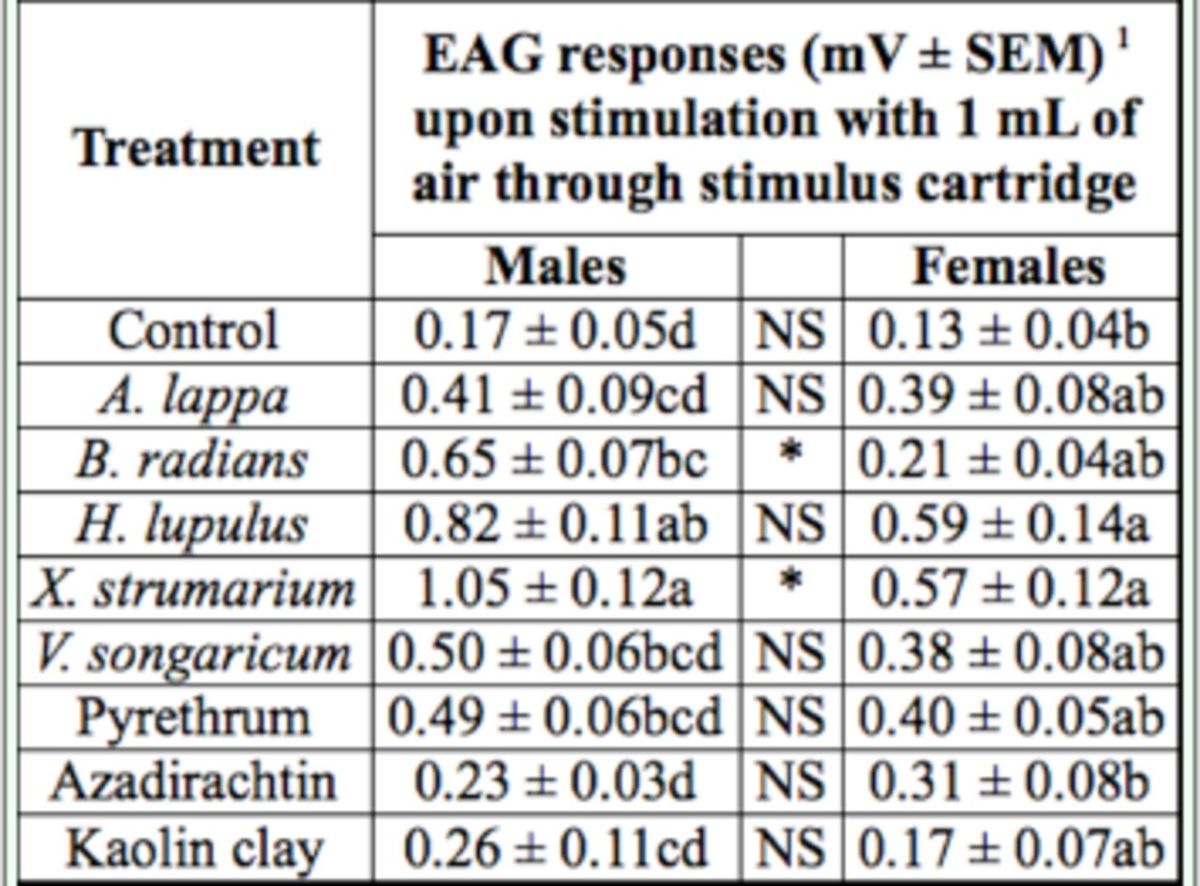
Electroantennogram responses of male and female plum curculio to various plant extracts and insecticides.

1Means within columns followed by the same letter are not significantly different ( *P* > 0.05, Tukey’s multiple comparisons test). Paired values within rows marked with an asterisk are significantly different ( *P* < 0.05) and NS indicates lack of significance.

**Figure 1.**
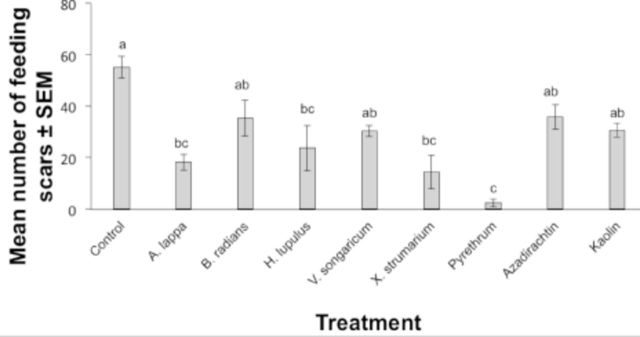
Mean number of feeding scars on apples treated with plant extracts, insecticides, or an untreated control. Bars indicated by the same letter are not significantly different (ANOVA followed by Tukey’s test, *P* > 0.05). Error bars indicate ± standard error of the mean (SEM). High quality figures are available online.

Extract and insecticide treatment significantly affected the combined feeding response of both sexes of plum curculio as compared with the control (F = 9.04; df = 8, 8; *P* < 0.01) ( Figure 1 ). There were significantly more feeding scars on control apples than on many of the treatments tested, with the exception of *B. radians* , *V. songaricum* , azadirachtin, and kaolin *.* Pyrethrum appeared to be the most effective treatment, resulting in approximately fewer than three feeding scars per apple. *X. strumarium* , *A. lappa* , and *H. lupulus* extracts also significantly reduced the number of feeding scars as compared with the control ( *P* < 0.05).

**Figure 2.**
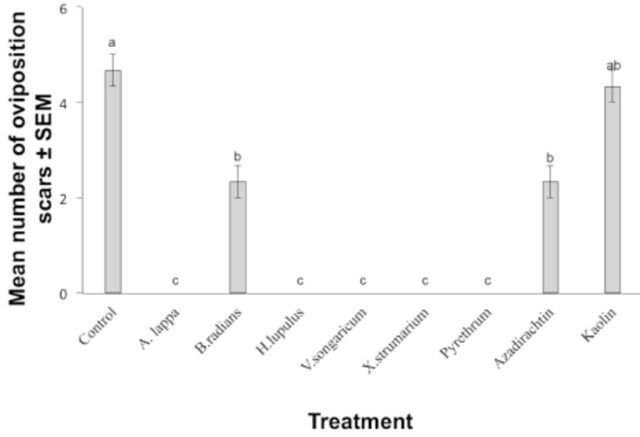
Mean number of oviposition scars on apples treated with plant extracts, insecticides, or an untreated control. Bars indicated by the same letter are not significantly different (ANOVA followed by Tukey’s test, *P* > 0.05). Error bars indicate ± standard error of the mean (SEM). High quality figures are available online.

The tested plant extracts and insecticide positive controls also affected oviposition behavior of plum curculio females ( Figure 2 ). Females laid fewer eggs on treated apples than on controls, and there was a significant difference between treatments (F = 24.25; df = 8, 18; *P* < 0.01) ( Figure 2 ). Interestingly, apples treated with kaolin clay received significantly more oviposition scars than many of the other chemicals tested and did not differ from the control treatment ( Figure 2 ). Plum curculio females did not lay any eggs into apples treated with *A. lappa* , *H. lupulus* , *V. songaricum* , or *X. strumarium* extracts, or those treated with pyrethrum, during the 72 hr incubation period ( Figure 2 ).

**Figure 3.**
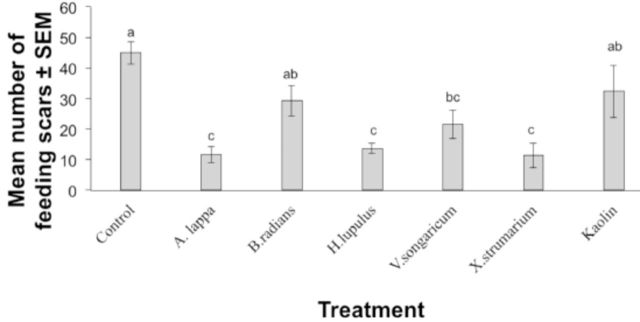
Mean number of feeding scars for each compound-ka olin clay treatment mixture versus an untreated negative control and the kaolin clay positive control in a no-choice assay. Bar s indicated by the same letter are not significantly different (ANOVA followed by Tukey’s test, *P* > 0.05). Error bars indicate ± standard error of the mean (SEM). High quality figures are available online.

The mixtures of plant extracts with kaolin clay significantly affected the number of feeding scars observed on apples (F = 12.11; df = 6, 14; *P* < 0.01) ( Figure 3 ). The number of feeding scars observed on apples treated with *X. strumarium* –kaolin clay, *A. lappa* –kaolin clay, and *H. lupulus* –kaolin clay mixtures were significantly lower than all other extracts, with the exception of the *V. songaricum* –kaolin clay mixture ( Figure 3 ). Although the *B. radi-*

*ans* –kaolin clay mixture reduced the number of feeding scars, this effect was not significantly different from the control or kaolin clay treatments ( *P* > 0.05).

**Figure 4.**
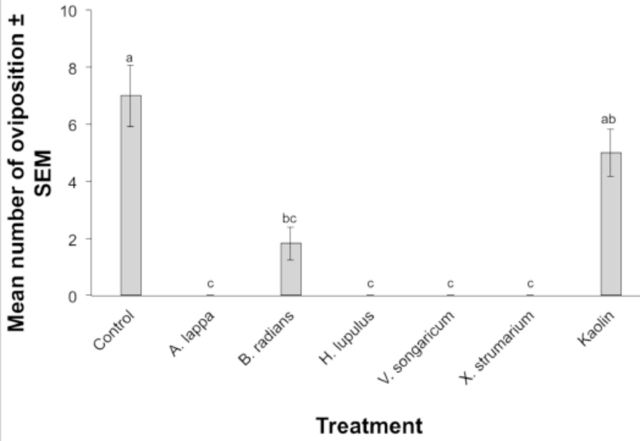
Mean number of oviposition scars for each plant extract-kaolin clay treatment mixture versus an untreated negative control and the kaolin clay positive control in a no-choice assay. Bars indicated by the same letter are not significantly different (ANOVA followed by Tukey’s test, *P >* 0.05). Error bars indicate ± standard error of the mean (SEM). High quality figures are available online.

Oviposition behavior of plum curculio was significantly affected by the plant extract– kaolin clay mixtures (F = 17.36; df= 6,14; *P* < 0.01) ( Figure 4 ). Among the tested plant extract–kaolin clay mixtures, *A. lappa* , *H. lupulus* , *V. songaricum* , and *X. strumarium* – kaolin clay mixtures produced the greatest oviposition-deterring effects, given that females did not lay any eggs on apples that received these treatments ( Figure 4 ). The total number of eggs laid by females on the apples treated with the *B. radians* –kaolin clay mixture was also significantly lower as compared with that on the control and kaolin clay treatments ( Figure 4 ).

### Discussion

Plum curculio monitoring techniques employ both trap-based ( Pinero et al. 2001 ; Prokopy et al. 2003 ; Leskey and Wright 2004 ) and trap tree-based ( Prokopy et al. 2003 ) systems in a limited array of commercial orchards. Grandisoic acid, benzaldehyde, and plum essence are the most effective chemicals used to attract plum curculio in orchards ( Coombs 2001 ; rPinero et al. 2001 ; Prokopy et al. 2001). While benzaldehyde is used as a synergist, grandiosoic acid is the principal pheromone of plum curculio for capturing adults. These chemicals are very effective in some situations for mass trapping plum curculio early in the season, yet this strategy wanes in effectiveness after fruit set ( Prokopy et al. 2003 ; Leskey and Wright 2004 ). This may be related to developing fruit chemicals that mask or out-compete both grandisoic acid and benzaldehyde and thus lead to declining or reduced behavioral response to traps. While current monitoring methods permit accurate scheduling of plum curculio control before fruit set, this masking phenomenon prevents the timely application of control strategies against plum curculio adults at other times of the year ( Reissig et al. 1998 ). Therefore, novel chemicals that may attract more plum curculio adults than the grandiosoic acid–benzaldehyde mixture or that inhibit the response of plum curculio to host plant volatiles could be useful tools for increasing the year-long effectiveness of trap-based monitoring and perhaps trap-out strategies or management through repellency.

Our behavioral and electroantennogram results indicate that some of the plant extracts examined herein may have potential application for “push-pull” management of plum curculio. The electroantennogram data confirm that plum curculio are capable of detecting the odorants released by these plant extracts at the level of the peripheral nervous system. However, a positive response does not confirm that the volatiles from the treatments tested caused repellency due to an olfactory mode of action. The extracts may mask host plant volatiles or directly repel plum curculio and cause adults to leave trees, especially after fruit set (“push”); attractants may serve as the “pull” or increase effectiveness of current monitoring techniques. It is possible that the mode of action of the plant-based extracts tested here includes repellency, as well as an-tifeedant and anti-oviposition effects. Therefore, the “push” force may be comprised of multiple effects on insect behavior. Determining the specific mechanism(s) that modify plum curculio behavior with these plant extracts will be important in our subsequent research. However, previous experiments suggest both repellency and oviposition deterrence as modes of action of these plant-based extracts ( Gökçe et al. 2005 , 2006a, 2006b, 2010).

The push strategy could also be effective against overwintering adults. Plum curculio move from overwintering sites to host trees in early spring and aggregate in border trees, where most of the damage occurs. Chemicals that may prevent host plant location or repel plum curculio from hosts should prevent colonization of cultivated crops and may increase the effectiveness of border row mass trapping. Mass trapping of overwintering adult plum curculio beyond the border rows of cultivated apple has been as effective as insecticide treatments in organic orchards (Whalon; unpublished data). Therefore, the extracts tested here may further improve the effectiveness of such mass trapping when used as complementary repellents.

The repellency effects of the extracts tested here have two potential additional benefits: reducing fruit injury and reducing damage from oviposition and larval development. In terms of fruit injury, both male and female plum curculio are more attracted to plant volatiles released by damaged fruits as compared with non-injured fruit ( Leskey and Prokopy 2001 ). In this study, the extracts tested inhibited feeding by both male and female plum curculio. Therefore, it is suggested that these extracts may aid in reducing initial feeding of plum curculio, potentially ensuring that fruit does not become more attractive to successive feeding. However, this hypothesis requires further testing.

In terms of oviposition and larval damage, these activities are of greatest threat in the spring, when overwintering adults migrate into the commercial orchard canopy. The oviposition-deterring effects of the extracts tested here have the potential to suppress this damage. Of particular importance is that some of the extracts completely inhibited oviposition by plum curculio in a laboratory setting. As plum curculio larval damage is essentially absolute (any damage is total loss of marketability), the demonstrated complete inhibition of oviposition by the extracts has the potential to greatly reduce loss of fruit and lower chances of violating fruit quality standards. This suppressive activity observed in the lab will require field verification.

In summary, the repellent and antifeedant effects of selected noxious plant extracts and known insecticides of plum curculio were successfully demonstrated in laboratory conditions. The results have particular implications for improving the effectiveness of the plant extracts by admixing with kaolin clay, with the potential impact of reducing plum curculio feeding and inhibiting oviposition. Isolation and characterization of active compounds in the extracts that exhibited behavioral and antennal activity is ongoing. It is hoped that future field verification of these formulations may produce a new tool for management of plum curculio.

## Abbreviations

AZM: azinphos-methyl
